# Doppler signal analysis of the tricuspid valve in healthy fetuses during the first trimester: a cohort study

**DOI:** 10.61622/rbgo/2026rbgo101

**Published:** 2026-02-20

**Authors:** Gustavo Fonseca de Albuquerque Souza, Cínthia Freire Carvalho, Adricia Cristine Souza Alves, Laura Mendes Rodrigues, Renato Barros Moraes, Alex Sandro Rolland Souza

**Affiliations:** 1 Instituto de Medicina Integral Prof. Fernando Figueira Centro de Atenção à Mulher Recife PE Brazil Centro de Atenção à Mulher, Instituto de Medicina Integral Prof. Fernando Figueira, Recife, PE, Brazil.; 2 Universidade Católica de Pernambuco Escola de Saúde e Ciências da Vida Recife PE Brazil Escola de Saúde e Ciências da Vida, Universidade Católica de Pernambuco, Recife, PE, Brazil.; 3 Universidade Federal de Pernambuco Centro de Ciências Médicas Recife PE Brazil Centro de Ciências Médicas, Universidade Federal de Pernambuco, Recife, PE, Brazil.

**Keywords:** Doppler ultrasound, Fetal heart, Prenatal diagnosis, Tricuspid valve, Pregnancy trimester, first

## Abstract

**Objective:**

This study aimed to characterize tricuspid valve sound signals in healthy fetuses during the first trimester of pregnancy using Doppler ultrasound and computational analysis.

**Methods:**

A cohort of pregnant women at 11–14 weeks of normal-risk gestation was assessed at the *Instituto de Medicina Integral Prof. Fernando Figueira* (IMIP) between December 2019 and May 2020. Eligible participants were over 18 years old with no pregnancy complications. Doppler recordings of the fetal tricuspid valve were obtained, and linear (wave duration) and non-linear analyses were performed, including approximate entropy (ApEn), Lempel-Ziv complexity (LZC), and detrended fluctuation analysis (DFA). Follow-up in the second trimester with a fetal medicine team and postnatal follow-up with physical examination by an experienced neonatology team confirmed normal cardiac results.

**Results:**

Early diastolic (E) wave presented a mean duration of 70.89±9.26 ms, with ApEn 0.25±0.15, LZC 0.77±0.15, and DFA 0.73. Atrial contraction (A) wave had 79.77±7.56 ms, ApEn 0.25±0.15, LZC 0.77±0.19, and DFA 0.52. Systole showed 225.95±15.88 ms, ApEn 0.15±0.17, LZC 0.78±0.15, and DFA 0.75. Diastole had 150.66±13.99 ms, ApEn 0.18±0.11, LZC 0.83±0.22, and DFA 0.66. The full cardiac cycle lasted 376.61±16.40 ms, with ApEn 0.18±0.11, LZC 0.83±0.22, and DFA 0.81. The diastole/cardiac cycle ratio was 0.4±0.3, with ApEn 0.22±0.12, LZC 0.72±0.15, and DFA 0.62.

**Conclusion:**

This study offers a detailed characterization of tricuspid valve wave segments in healthy fetuses during early gestation. The integration of linear and non-linear analyses may enhance our understanding of fetal cardiac physiology. Further research is needed to evaluate whether these parameters can assist in the early detection of congenital heart diseases. We highlight the potential of these parameters for early screening of congenital heart disease and chromosomal abnormalities, while emphasizing the need for further studies to confirm their diagnostic value.

## Introduction

Congenital malformations are functional, structural, or metabolic abnormalities occurring from embryonic development to birth and are the second leading cause of mortality in children aged under one year.^(1)^ In Brazil, congenital heart disease (CHD) is the most common postnatal malformation, resulting in increased morbidity and mortality rates in the first year of life.^(2)^ CHD affects approximately 0.9% of live births; 20% to 30% have severe structural defects, and of these, 3% to 5% die in the neonatal period.^(1)^ Because cardiac development occurs mainly up to the eighth week of gestation, early prenatal diagnosis is essential to optimize fetal outcomes and to prepare the family for birth.^(1,3,4)^

Investigation methods for CHD include the tricuspid valve assessment. The systemic tricuspid valve regurgitation is often related to CHD (e.g., cardiomegaly, Ebstein's disease, pulmonary atresia) and can be the cause or consequence of right ventricular dysfunction.^(5)^ Although tricuspid valve regurgitation is a marker of aneuploidy in the morphological scan of the first trimester of pregnancy, it can be found in 1% of euploid fetuses.^(6)^ In a study with 19,614 patients, tricuspid valve regurgitation was observed in 56% of fetuses with Down syndrome and 33% of those with Patau, Edwards, and Turner syndromes.^(5-7)^

The use of Doppler velocimetry in tricuspid valve evaluation may contribute to the understanding of CHD, yet studies on this subject remain scarce.^(5-8)^ Advances in ultrasound technology have improved the ability to identify subtle anatomical and physiological cardiac changes during pregnancy, particularly in the first trimester.^(9)^ In addition to conventional (linear) Doppler analysis, non-linear computational methods such as approximate entropy (ApEn), Lempel-Ziv complexity (LZC), and detrended fluctuation analysis (DFA) have emerged as promising tools for characterizing cardiovascular patterns, providing a deeper understanding of fetal cardiac physiology.^(8-15)^

Establishing reference values for these parameters in normal fetuses represents a necessary first step before applying them in high-risk populations, such as those with aneuploidy or structural heart disease. In this context, the present study aimed to characterize tricuspid valve sound signals in healthy fetuses during the first trimester, using Doppler ultrasound and both linear and non-linear computational analysis methods.

## Methods

This prospective cohort study recruited normal-risk pregnant women using convenience and consecutive sampling through medical records of those who underwent Doppler ultrasound in the fetal medicine sector of the *Instituto de Medicina Integral Prof. Fernando Figueira* (IMIP) from December 2019 to May 2020.

Pregnant women in the first trimester (i.e., 11 to 14 weeks) presenting a single, topical, and normal-risk pregnancy were included. Those aged under 18 years or with a diagnosis of aneuploidies or congenital malformations were excluded.

Women characteristics were collected using a form in the ultrasound room, and the Doppler velocimetry was conducted in the first trimester to detect aneuploidies and congenital malformations. Morphological scan was conducted in the second gestational trimester to detect congenital malformations. Women records were assessed after birth to collect postnatal data, and those who delivered in another institution were contacted by telephone to respond the postnatal form.

Prenatal variables were maternal age (years), skin color, gestational age (weeks), number of previous deliveries, smoking habits, comorbidities diagnosed during pregnancy (e.g., diabetes mellitus, hypertensive disease), nuchal translucency, and body mass index (BMI, kg/m^2^). Postnatal variables included gestational age at birth (weeks), birth weight (grams), sex, type of delivery (vaginal or cesarean), Apgar score in the first and fifth minute, and congenital malformations identified at the first physical examination. The variables of sound analysis were linear spectroscopy of the tricuspid valve, such as the duration of the E (rapid ventricular filling) and A (slow ventricular filling) waves, total diastole, systole, cardiac cycle, diastole/cardiac cycle (D/C) ratio, and instantaneous fetal heart rate (FHR). Non-linear parameters of computational analysis included ApEn, LZC, and DFA of the E and A wave, total diastole, systole, cardiac cycle, and D/C ratio.

A specialist in fetal medicine conducted the morphological scan in the first and second gestational trimesters following the recommendations of the International Society of Ultrasound in Obstetrics and Gynecology (ISUOG) and the Fetal Medicine Foundation (FMF).^(8,16)^ During the assessment, women remained in the supine position with an empty bladder, and a convex transducer (3 to 6 MHz) coupled to the Voluson S8 ultrasound device (Samsung Medison, Gyeonggi-do, South Korea) was used. The assessments lasted about 30 minutes. In the first trimester, fetal morphology, biometry, measurement of nuchal translucency and nasal bone, assessment of the ductus venosus pulsatility index, and tricuspid valve velocimetry were performed.^(8,16)^ Any change in these parameters was considered positive screening for aneuploidy, according to FMF reference values.^(8,16)^

It is important to emphasize that the exams were always performed in the same room, with a standardized ambient temperature, to minimize external interference. Furthermore, the participants’ medical history was obtained at the time of the exam by research staff, including comorbidities, medication use, current symptoms, and other complaints, enabling the identification of maternal conditions that could impact the measurements.

The same device was used for Doppler velocimetry and blood flow was obtained using the triplex system (i.e., two-dimensional image, color Doppler, and pulsed Doppler) and color mapping coupled to a 3.75 MHz convex transducer. Women were maintained in a semi-Fowler position to avoid postural hypotension during assessments. The acoustic power of the Doppler mode with color mapping was maintained below the standards established by the Food and Drug Administration and International Federation of Gynecology and Obstetrics for fetal ultrasound, with an average spatial-temporal peak intensity of 57 mW/cm^2^.^(8)^

The Doppler ultrasound of the tricuspid valve was performed by visualizing the fetal heart and maximizing the thorax image on the screen. The sample volume was between two and three millimeters, positioned vertically, and with an angle of insonation below 30^o^. The heart was observed in an apical position, with the fetal back in an anterior or posterior position. The ultrasound focus was placed on the center of the tricuspid valve for one to two minutes, three to five consecutive times, prioritizing ultrasounds with less fetal activity to avoid movement artifacts.^(8,16)^ A half systole and a velocity above 60 cm/s characterized tricuspid valve regurgitation in the first trimester.

Sound signals were extracted from ultrasound videos and recorded in wave format (.wav) at a sampling rate of 48kHz.^(8)^ The tricuspid valve data from Doppler ultrasound were recorded on a USB stick during the assessment. Next, the video files (.mpg) were extracted to a computer, and audios were extracted from the videos in wave format (.wav) using a website (https://convertio.co/pt/mpg-wav/) to filter and measure the wave intervals.

**Figure 1:**
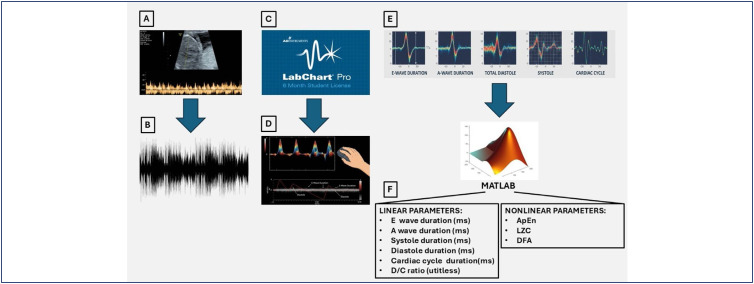
Doppler signal processing of the fetal tricuspid valve for linear and nonlinear parameter analysis

Two independent and previously trained researchers segmented 30 cardiac cycles in the LabChart software version 7.3.8 (ADInstruments, Dunedin, New Zeland), discarding those presenting artifacts as a way to reduce intraobserver and interobserver variability. Next, they transferred the data to an Excel spreadsheet version 2016 (Microsoft Corp, Washington, USA), including instantaneous FHR, duration of E and A waves, total diastole, systole, cardiac cycle, and D/C ratio. The initial and final periods of the ultrasound were used to extract the segments corresponding to the E and A waves, diastole, systole, cardiac cycle, and D/C ratio from the original audio file (.wav) in the QuB software version 1.4.0.1000 (MLab Edition, University of Missouri, USA). Sampling rates were preserved, and the active ultrasound recording channel was used. After selection, the duration intervals of the tricuspid valve waves were loaded into the MATLAB software version 2017a (Mathworks Inc., Massachusetts, EUA) and submitted to the ApEn, DFA, and LZC algorithms (adjusted for the analyzed data), website (https://physionet.org/) (Figure 1).

A- Original ultrasound video; B- Audio extracted from the video; C- Labchart (program) used to select and extract the spectral signal; D- Manual signal selection and segmentation; E- Each of the segments used, for both linear and nonlinear analysis; F- Results obtained from running the algorithms in MATLAB

The ApEn is a non-linear method for investigating the complexity and regularity of signals with different lengths from a minimum series, using 15% tolerance of the standard deviation (SD) of the series to compare the minimum series and its increasing number with the entire series. Recently, ApEn has been used to assess heart rate variability.^(11,12,17)^

The DFA algorithm verifies a long-range correlation in a non-stationary time series. DFA is initially integrated into the series (dividing it into boxes), and a trend is calculated for each box, followed by the deviations of the series. Finally, it verifies whether the size of the boxes and their trend value present a linear correlation. Values indicate random data in the series (0.5), negative correlation (< 0.5), or positive correlation (0.5 to 1).^(18)^

The LZC was adapted to assess the entropy of time series in samples of different types and is a suitable alternative for the Lyapunov exponent to assess biomedical signals. It can be performed by establishing a threshold to binarize the series, and the variation size in the data series can be established with a minimum sequence of characters. Also, LZC can be used for small and large series with low computational power demand, facilitating software development to classify series with small data.^(19)^

Statistical analysis was performed using the OriginPro software version 8.0 (OriginLab Corp., Massachusetts, USA) to calculate measures of central tendency and dispersion for numerical variables and frequency distribution for categorical variables. This study is exploratory and descriptive, without inferential statistical analyses. As a pioneering investigation of first-trimester fetal tricuspid valve wave segments, it establishes baseline data and serves as a foundation for future studies in larger and higher-risk populations.

The study was approved by the research ethics committee of the IMIP (approval no. 12873719.0.0000.5201, opinion no. 3.780.813) and followed the Declaration of Helsinki. All participants signed the informed consent form.

## Results

A total of 78 pregnant women were eligible for this study; five did not agree to participate, and six presented congenital malformations after ultrasound assessment. Also, 12 women did not return for assessments in the second gestational trimester or could not be contacted after birth, and four miscarried. Therefore, 51 pregnant women participated in this study. The mean maternal age was 29.4 ± 6.3 years, and the mean gestational age was 12.7 ± 0.7 weeks. Most women presented brown skin color 35(68.6%), were non-smokers 46(90.2%), and had no comorbidities 38(74.5%). The mean BMI was 27.8 ± 7.0 kg/m^2^, and the mean number of previous deliveries was 1.2 ± 1.4. The mean instantaneous FHR in the ultrasound was 160.8 ± 6.8 bpm (Table 1).

**Table 1:** Epidemiological profile of pregnant women

Prenatal variables		mean ± SD
Maternal age, years		29.4 ± 6.3
BMI, kg/m^2^		27.8 ± 7.0
Gestational age, weeks		12.7 ± 0.7
Instantaneous fetal heart rate		160.8 ± 6.8
Nuchal translucency		2.00 ± 2.1
Previous deliveries		1.2 ± 1.4
**Prenatal variables**		**n(%)**
Skin color		
	Brown	35(68.6)
	Black	10(19.6)
	White	4(7.8)
	Other	2(4.0)
Smoking habit		
	No	46(90.2)
	Yes	5(9.8)
Comorbidities		
	No	38(74.5)
	Yes	13(25.5)

SD: standard deviation. BMI: body mass index

In the postnatal variables, the mean gestational age at birth was 38.6 ± 1.8 weeks, and cesarean was the most prevalent type of delivery 13(68.4%). Most newborns were male 30(58.8%) and had Apgar scores of 8.4 ± 0.8 and 9.6 ± 0.5 in the first and fifth minutes, respectively. Also, none of the newborns showed congenital malformation after birth (Table 2).

**Table 2:** Epidemiological profile of newborns

Postnatal variables		mean ± SD
Gestational age at birth, weeks		38.6 ± 1.8
Apgar in the first minute		8.4 ± 0.8
Apgar in the fifth minute		9.6 ± 0.5
Birth weight (grams)		3,225.9 ±5 75.8
**Postnatal variables**		**n(%)**
Type of delivery		
	Cesarean	35(68.4)
	Vaginal	16(31.6)
Sex		
	Male	30(58.8)
	Female	21(41.2)
Congenital malformations		
	No	51(100.0)
	Yes	0(0)

SD: standard deviation

The following mean duration was observed in the linear methods: 70.89 ± 9.26 ms for E waves, 79.77 ± 7.56 ms for A waves, 225.95 ± 15.88 ms for systoles, 150.66 ± 13.99 ms for diastoles, 376.61 ± 16.40 ms for cardiac cycles, and 0.40 ± 0.30 ms for D/C ratio. After applying non-linear methods, the E wave of the tricuspid valve presented an ApEn of 0.25 ± 0.15, LZC of 0.77 ± 0.15, and DFA of 0.73. The A wave presented an ApEn of 0.25 ± 0.15, LZC of 0.77 ± 0.19, and DFA of 0.52, and systole presented an ApEn of 0.15 ± 0.17, LZC of 0.78 ± 0.15, and DFA of 0.75. The diastole presented an ApEn of 0.18 ± 0.11, LZC of 0.83 ± 0.22, and DFA of 0.66, cardiac cycle presented an ApEn of 0.18 ± 0.11, LZC of 0.83 ± 0.22, and DFA of 0.81, and the D/C ratio presented an ApEn of 0.22 ± 0.12, LZC of 0.72 ± 0.15, and DFA of 0.62 (Table 3).

**Table 3:** Linear and non-linear analysis of the fetal tricuspid valve sound signals during the first trimester of pregnancy

Variables	Duration (ms) (mean ± SD)	ApEn (mean ± SD)	LZC (mean ± SD)	DFA
E wave	70.89 ± 9.26	0.25 ± 0.15	0.77 ± 0.15	0.73
A wave	79.77 ± 7.56	0.25 ± 0.15	0.77 ± 0.19	0.52
Systole	225.95 ± 15.88	0.15 ± 0.17	0.78 ± 0.15	0.75
Diastole	150.66 ± 13.99	0.18 ± 0.11	0.83 ± 0.22	0.66
Cardiac cycle	376.61 ± 16.40	0.18 ± 0.11	0.83 ± 0.22	0.81
D/C ratio	0.40 ± 0.3	0.22 ± 0.12	0.72 ± 0.15	0.62

DFA = detrended fluctuation analysis; ApEn = approximate entropy; LZC = Lempel-Ziv complexity; SD = standard deviation; E wave = rapid ventricular filling; A wave = slow ventricular filling; D/C ratio = diastole/cardiac cycle ratio

## Discussion

Understanding fetal, maternal, and placental physiology is essential in the study of fetal development and cardiovascular assessment during intrauterine life.^(19,20)^ The heart is one of the first organs in embryonic development, with evident cardiac movement between the fifth and sixth weeks of pregnancy. Also, the tricuspid valve is the last cardiac structure to be developed (ninth and tenth weeks of pregnancy), located between the atrium and the right ventricle, and essential to maintain the direction of cardiac blood flow.^(21-23)^

The functioning of the tricuspid valve can be measured using pulsed Doppler. During the isovolumetric contraction in early embryonic life, blood flow passes from the right atrium to the right ventricle through the tricuspid valve in the ventricular diastole only by atrial contraction. However, the rapid ventricular filling phase begins before atrial contraction due to the developed ventricular relaxation from the ninth week of pregnancy.^(24)^

The E wave corresponds to rapid ventricular filling and can be identified after the tricuspid opening using ultrasound, while the A wave originates during atrial contraction when the blood flows fast through the valve. The flow is monophasic when only the A wave is identified and biphasic when the A and E waves are identified.^(25)^ Also, adequate fetal cardiac compliance is identified from the second gestational trimester, characterized by an increase in early diastolic velocity (E wave) and decrease in the E/A waves ratio.^(26)^

Blood flow regurgitation may occur through the tricuspid valve during systole in the first trimester, being a functional phenomenon in most cases that may appear in 0.9% of euploid fetuses.^(7)^ However, tricuspid valve regurgitation is diagnosed when reflux is observed during at least half of the systole with a velocity > 80 cm/s since aortic and pulmonary blood flow produces a maximum velocity of 50 cm/s.^(6,26)^ Also, this dysfunction can be found in CHD (e.g., atrioventricular septal defects, Ebstein's anomaly, and pulmonary atresia with intact ventricular septum) and trisomy 13, 18, and 21.^(7,27)^

A prospective study of transabdominal fetal cardiac assessment using a high-frequency linear transducer showed the association between tricuspid valve regurgitation and CHD, identifying tricuspid valve abnormalities in 61.5% of euploid fetuses with CHD *versus* 9.2% of those without cardiac abnormalities. Thus, cardiac hemodynamics should be assessed using different methods, allowing them to become early diagnostic tools for CHD or even screening methods for aneuploidies. Ninno^(10)^ assessed the tricuspid valve flow using Doppler velocimetry in fetuses from 11 to 13 weeks and 6 days of pregnancy, and the duration of cardiac cycle (390 ± 21.1 ms), diastole (147 ± 18.0 ms), and D/C ratio (0.38 ± 0.04) were similar to those observed in the present study.^(10)^

An important point to note is the relatively high dispersion observed in the diastole/cardiac cycle (D/C) ratio (0.40 ± 0.30). This variability may reflect intrinsic differences in early fetal cardiac physiology, since diastolic function at this gestational stage is still maturing and may be more susceptible to physiological fluctuations. Therefore, the interpretation of the D/C ratio should be made with caution, and further studies with larger cohorts are needed to better define its reference range.

In a pioneer study, the maximum atrioventricular flow velocities were analyzed simultaneously (i.e., mitral and tricuspid valves), with E wave values of 20.5 ± 3.2, A wave of 38.6 ± 4.7, and A/E ratio of 0.53 ± 0.05.^(28)^ After a year, the same research group analyzed the maximum flow velocity specifically for the tricuspid valve and found a mean velocity of 9.7 ± 2.1 cm/s, E wave of 23.1 ± 4.9 cm/s, and A wave of 41.9 ± 5.3 cm/s.^(29)^ Notably, the maximum flow velocity was assessed using a linear analysis.

Several non-linear methods (e.g., ApEn, LZC, and DFA) are being developed to facilitate data analysis from fetal Doppler ultrasound. The ApEn calculation depends on the number of data points, embedding dimension, and tolerance, and this method has been used to analyze physiological time series, mainly from the cardiovascular system.^(17)^ This method has also been used to analyze physiological time series, mainly from the cardiovascular system. Considering that it quatifies regularity in data, high ApEn values are directly related to complex and irregular signals.^(11,12)^ Thus, a vulnerable connection within a system indicates the presence of the disease mechanism, increasing the regularity of the series.^(14)^ The ApEn is one of the essential methods to study fetal heart rate variability, which is closely related to cardiovascular events, and is a powerful tool for understanding the pathological process by comparing its non-linear characteristics with the physiological phenomena.^(11,12)^ The present study found ApEn values ranging from 0.15 to 0.25, which corroborated the theory of physiological cardiovascular stability in fetuses without changes in the tricuspid valve.

The LZC can calculate data collected at regular time intervals (i.e., time series) even without long segments of this data, and a different pattern is obtained in time series according to the signal type, which can be divided into recurrent (low complexity) and individual (high complexity).^(14)^ It transforms an analyzed signal into a binary sequence, allowing it to quantify the rate of new patterns and increasing the complexity value.^(15)^ The LZC has excellent application in the analysis of biosignals, detecting events (e.g., epileptic seizures, onset of ventricular tachycardia, fibrillation, and changes in the sleep-wake cycle) and allowing the study of DNA sequences.^(30,31)^ Also, it is a reliable method for analyzing the differentiation of time series of variability in the cardiovascular system.

Similar to the ApEn, the LZC can be used to assess fetal heart rate variability and detect changes in neonatal distress (e.g., intrauterine growth restriction), highlighting the importance of non-linear methods to monitor fetuses. Also, the benefit of these measures involves the interpretation of signal processing, assessment of development and gestational age, and fetal well-being.^(14)^ A comparative study between FHR signals and complexity parameters identified an LZC value of 0.94 in fetuses with severe intrauterine growth restriction, indicating a random sample, whereas healthy fetuses presented mean values < 0.9.^(31)^ According to the authors, LZC is a stable parameter that differentiates severe from moderate intrauterine growth restriction and healthy fetuses.^(31)^ These results corroborated those found in the present study, in which LZC values ranged between 0.72 and 0.83 in healthy fetuses.

This study represents a pioneering approach in the first trimester of pregnancy, applying a multiparametric analysis that combines linear and non-linear parameters. This characterization provides a baseline for future research aiming to expand the understanding of early fetal cardiac physiology.

The DFA was another measure addressed, characterized by fractal and non-linear analysis (i.e., quantification of intrinsic correlation properties [short and long-range] of dynamic systems through DFA parameters). Also, it can detect intrinsic self-similarity incorporated into a non-stationary time series and avoid the detection of apparent self-similarity.^(18,32)^ Therefore, the fetal heart rate variability can be analyzed using this measure since the variation occurs in different time scales, being similar to itself.^(29)^

The DFA for the duration of wave segments of the tricuspid valve ranged between 0.52 and 0.81, indicating a long-range memory between series during the first trimester. Researchers have been using DFA analysis to identify whether patients with acute myocardial infarction or congestive heart failure have an acute or chronic condition.^(33,34)^ It is based on the theory that individuals with regular cardiac function present a vagal response after increased sympathetic activity to control heart rate variability, whereas pathologies would lead to physiological dysfunctions and changes in DFA values.^(35)^

Despite these promising findings, the direct clinical interpretation of ApEn, LZC, and DFA remains at an early stage. Longitudinal studies and investigations in high-risk populations are needed to determine whether these parameters can reliably distinguish pathological from physiological variability. An additional perspective is the potential integration of these computational parameters into first-trimester combined screening protocols. For example, future studies could evaluate their additive value alongside established markers such as nuchal translucency (NT), ductus venosus pulsatility index (DV PI), and qualitative tricuspid regurgitation.

Another relevant aspect is the potential for standardization and automation of computational analyses such as ApEn, LZC, and DFA. With advances in signal processing and machine learning, it may become feasible to incorporate these techniques into routine ultrasound assessment, reducing operator dependency and allowing objective, reproducible evaluation of fetal cardiac physiology. This standardization could facilitate broader application in clinical practice and enhance the value of multiparametric approaches for early screening.

Recent research has advanced the application of nonlinear and computational analyses in fetal cardiovascular assessment, particularly in studies exploring heart rate variability, complexity indices, and predictive models for fetal acidosis and hypoxia.^(14,35-37)^ These approaches demonstrate the growing relevance of nonlinear methods for understanding fetal physiology. However, to date, no studies have specifically applied such techniques to Doppler signals analysis of the fetal tricuspid valve in early gestation. This gap underscores the originality of our findings and highlights the importance of developing reference values and methodologies that may expand the use of these analyses to improve fetal cardiac evaluation.

The main limitation of this study was the loss to follow-up during prenatal care and the sample loss during contact with women for postnatal data collection. We emphasize that part of this loss is due to the socioeconomic and cultural conditions of patients in the Northeast region of Brazil, who often face difficulties returning to the health service, which partly explains the percentage distributed. Also, memory bias might have affected the data from women who did not deliver at the institution. Another limitation is related to signal acquisition. Extracting sound through .wav files from ultrasound video may reduce signal fidelity, potentially affecting the precision of computational analyses. Although standardized acquisition protocols were applied, this factor should be considered when interpreting the results. However, this bias was minimized by asking them to verify their prenatal card or hospital discharge summary for responses. Newborns were considered healthy based only on physical assessment, which may have missed subtle congenital heart defects, as genetic testing and echocardiography were not routinely performed due to limited resources. However, all assessments were conducted by experienced neonatology specialists, even for newborns from other institutions, which might have minimized this limitation. Few studies have used these computational analyses to evaluate fetal physiology, specifically the tricuspid valve in the first trimester, reinforcing the importance of initial characterization of healthy fetuses to assess whether this methodology can be used to diagnose CHD and screen aneuploidies early. Furthermore, as this is an initial study, we highlight the limitation of not performing the sample size calculation.

## Conclusion

This study characterized the cardiovascular hemodynamics of healthy fetuses using Doppler ultrasound of wave segments of the tricuspid valve, and findings may serve as a basis for monitoring normal-risk pregnancies. A multiparametric approach should be conducted to identify changes in fetal cardiac physiology, and further studies should determine the validity of the parameters for this population, specifically in fetuses at risk, such as those with CHD. These findings may contribute to the development of novel, objective parameters for early screening of congenital heart disease and chromosomal abnormalities in the first trimester.

## Data Availability

The authors did not make the data from this article available in repositories prior to submission.
